# Diastolic Electrocardiographic Parameters Predict Implantable Device Detected Asymptomatic Atrial Fibrillation

**DOI:** 10.4274/balkanmedj.2016.0246

**Published:** 2017-09-29

**Authors:** Ahmet İlker Tekkeşin, Yalçın Velibey, Ceyhan Türkkan, Ahmet Taha Alper, Yasin Çakıllı, Tolga Sinan Güvenç, Ozan Tanık, Adnan Kaya, Özlem Yıldırımtürk, Nazmiye Özbilgin, Özge Güzelburç, Ahmet Öz, Regayip Zehir, Kadir Gürkan

**Affiliations:** 1 Department of Cardiology, University of Healty Sciences, Dr. Siyami Ersek Thoracic and Cardiovascular Surgery Training and Research Hospital, İstanbul, Turkey

**Keywords:** Diastolic electrocardiographic parameters, implantable cardiac device, atrial fibrillation

## Abstract

**Background:**

Atrial fibrillation is the most common clinically significant arrhythmia. It is now established that atrial high-rate episodes are highly correlated with atrial fibrillation.

**Aims:**

To investigate the relation between diastolic electrocardiographic parameters and subclinical atrial fibrillation detected by cardiac implantable electronic devices.

**Study Design:**

Ccross-sectional study.

**Methods:**

A total of 203 patients who had a dual-chamber, rate-modulated pacing pacemaker implanted due to sinus node dysfunction were prospectively enrolled in this study. Atrial high-rate episodes were defined as any lasting more than 5 min with an atrial rate of ≥220 beats per minute during the previous year. Patient groups were categorized on the basis of pacemaker interrogation as the absence of atrial high-rate episodes [atrial high-rate episodes (-)] and the presence of atrial high-rate episodes [atrial high-rate episodes (+)]. Episodes related to atrial over sensing were excluded. Twelve-lead surface electrocardiography was independently analyzed by two experienced readers for the measurement of diastolic electrocardiography parameters.

**Results:**

Among 203 patients (mean age: 67.5±9.1, 60.1% male), 51 (25.1%) with atrial high-rate episodes were defined as group 1 and 152 (74.9%) without atrial high-rate episodes were defined as group 2. Both groups were similar in terms of demographic characteristics and cardiovascular risk factors. Tend-Q and Tend-P were significantly longer in group 2. PQ interval was statistically longer in group 1. Corrected QT interval was significantly longer in group 1. Diastolic electrocardiography index, heart rate and PQ and QT intervals were the only independent predictors of atrial high-rate episodes in patients with dual pacemakers in multivariate analysis.

**Conclusion:**

Abnormal diastolic electrocardiography parameters are powerful predisposing factors for the initiation of incident atrial fibrillation. Diastolic electrocardiography parameters and a novel diastolic index predict atrial high-rate episodes. Evaluating these parameters enables clinicians to identify patients who are at high risk and who may benefit from prophylactic treatment.

Atrial fibrillation (AF) is one of the most common clinically significant arrhythmias. The association of AF with an increased risk of stroke is not dependent on whether it is clinically symptomatic (1). The risk of stroke is doubled in the presence of AF (2). Subclinical AF is proposed to be the underlying mechanism in patients with ischemic stroke of unknown etiology, which constitutes approximately 25% of total ischemic stroke events (3,4). Technological advances in pacemaker systems have enabled the detection, analysis and storage of atrial high-rate episodes (AHRE) during device interrogation, which in turn, has led to the emergence of the term “silent AF” (5). AHREs are surrogates for clinically asymptomatic AF and they have recently been demonstrated to be highly correlated with atrial flutter and AF, especially when AHRE are >5 min in duration (6). Therefore, AHRE are considered a significant indicator and precursor of AF. Several previous studies demonstrated that thromboembolism risk is associated with the total duration or burden of AF detected by cardiac implantable electronic devices (CIED) (7,8).

Diastolic dysfunction (DD) shares many common risk factors with AF, including age, hypertension (HT) (9), obesity (10) and diabetes (11). DD has deleterious effects on atrial function and structure, many of which are pro-arrhythmic. It has been shown that DD is a significant predisposing substrate for AF (12). There are several limitations in most non-invasive measurements of left ventricular relaxation, stiffness and filling pressures. These measurements are often established on simplified assumptions, which makes the probability of their general application low. Moreover, their assessment is highly dependent on hemodynamic parameters such as preload, afterload and sympathetic tone (13). The non-invasive measurements of left ventricular relaxation, stiffness and filling pressures are usually indirect. In addition, they are mostly based on simplified assumptions. For these reasons, they are considered to have limitations and are not generally applicable. Changing preload, afterload and sympathetic tone can affect their assessment, even in the same patient, complicating their measurement and interpretation (13). In contrast, hemodynamic changes have no impact on electrocardiographic (ECG) parameters. ECG parameters are operator-independent and show significant reproducibility (14). Diastolic ECG parameters such as PQ, Tend-P, Tend-Q and a combined novel ECG index consisting of age, PQ-interval and Tend-P [Tend-P/(PQ x age)] have relatively high accuracy in diagnosing DD (15). In the present study, we aimed to demonstrate the association between diastolic ECG parameters and subclinical AF detected by CIED.

## MATERIALS AND METHODS

### Study design and patient selection

This study was designed as cross-sectional study. We included patients who received a dual-chamber, rate-modulated pacing pacemaker, which was implanted at least 1 year earlier due to sinus node dysfunction (SND) such as sinus pause, Sick Sinus syndrome and intermittent sinus bradycardia. Between 1 January 2014 and 30 December 2014, a total of 250 patients were evaluated during routine pacemaker control. For an error margin of 5% and a power of 80%, the study sample that was needed to demonstrate statistical significance was calculated as 50 patients. Patients under the age of 18 years, patients with previous atrial arrhythmia, any degree of AV block, acute or chronic renal failure, any anti-arrhythmic medication use, congenital heart disease (including patients with a history of surgery for congenital heart disease), significant valvar heart disease, patients with electrolyte disorders and patients with pacemaker rhythm were excluded from the study. In addition, patients with atrial and/or ventricular pace rhythm on the ECG used for the measurement of diastolic parameters were also excluded. Pace rhythm was not present on the ECG of any patient included in this study, and patients with bradycardia (heart rate <60 beats per minute) or tachycardia (>90 beats per minute) on baseline ECG were excluded. Forty-seven patients were excluded based on the exclusion criteria, and data on 203 patients were found to be eligible for analysis. Patients’ medical histories were recorded, and a physical examination was performed. Demographic and clinical information was recorded for all patients at the time of enrollment. Diabetes mellitus was defined as previous diagnosis of diabetes, previous use of antidiabetic agents or as having at least two fasting blood sugar measurements >126 mg/dL during the examination. HT was defined as a systolic blood pressure ≥140 mmHg and/or a diastolic blood pressure ≥90 mmHg on at least two separate measurements during the examination, previous diagnosis of HT or previous use of antihypertensive medications.

Pacemakers were interrogated to detect AHRE. Patients were divided into two groups according to presence or absence of AHRE [AHRE (+) or AHRE (-), respectively]. AHREs were defined as atrial high rates faster that 220 bpm and lasting longer than 5 min on the basis of previous studies demonstrating their significance regarding increased rate of stroke and thromboembolic events (6). Termination of AF was defined as the occurrence of 20 beats below the AHRE detection rate to ensure the exclusion of short episodes of atrial premature beats. The atrial tachycardia detection mode was enabled, and the AF suppression by performing atrial overdrive pacing feature was programmed off. Bipolar atrial lead sensitivity and the post ventricular atrial blanking period were interrogated properly to reduce P-wave sensitivity and far-field R-wave over-sensing in order to identify atrial activities during AHRE. Atrial activity during AHRE was more easily detected by lowering P-wave sensitivities. We excluded patients who had abnormalities in atrial sensing. Laboratory tests, including blood chemistry, complete blood count and baseline 12-lead ECG were obtained from all patients. Serum blood urea nitrogen, creatinine and electrolyte levels were measured as part of the biochemical parameters using an Architect plus ci 4100, (Abbott Laboratories, Abbott Park, Illinois, USA). All subjects were evaluated in the transthoracic echocardiography (TTE) laboratory of our hospital. TTE evaluation was performed on each study patient by a cardiologist who was an expert in cardiovascular imaging. All measurements were made with the same probe on the same echocardiography device. Each patient was evaluated by M mode echocardiography. Left atrial and ventricular chamber diameters were measured according to chamber qualification guidelines by the American Society of Echocardiography. Left ventricular ejection fraction was measured as a part of two-dimensional echocardiographic examination performed on all patients using the biplane Simpson method. A 2.5-3.5 MHz phased-array transducer and a GE Vivid7 system (GE Vivid 7, GE Healthcare, Piscataway, NJ, USA) were used. The study was approved by the institutional ethics committee, and written informed consent was obtained from all patients.

### Electrocardiographic measurements

**FIG. 1.**
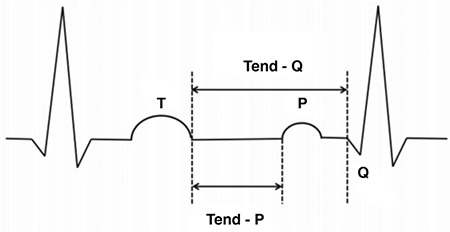
Schematic illustration of Tend-P and Tend-Q measurements.

Two experienced readers independently analyzed the 12-lead ECG tracings recorded at 25 mm/sec for the measurement of diastolic parameters and diastolic ECG index. The observers were blinded to the presence of AHRE. Standard criteria for ECG findings were applied: The QTc interval was calculated using the Bazett formula (16). Namdar et al. (15) investigated a potential role of ECG indices for the identification of patients with DD. Five parameters were found to be significantly correlated with the global assessment of diastolic function: age, heart rate, PQ, Tend-P and Tend-Q. Tend-P and Tend-Q were the only parameters to remain significant after adjustment for possible confounders, which were age, heart rate and PQ. On the other hand, these parameters did not show adequate diagnostic performance to be sufficient alone. The most relevant parameters were combined to generate two novel indices. In order to make a distinct differentiation of groups and to separate overlaps, the strongest single parameters (based on the above-mentioned analysis) were used in combination: Tend-P [Tend-P/(PQ x age)]. We also included in our analysis the ECG intervals which show the mechanical diastole (Tend-P, Tend-Q). We both manually measured (considering all ECG leads) and calculated (RR minus PQ minus QT for Tend-P and RR minus QT for Tend-Q) these two intervals. Diastolic ECG index was calculated as [Tend-P/(PQ x Age)] (Figure 1). Single leads with T waves smaller than 1.5 mm in amplitude were not included in the analysis.

### Statistical analysis

For statistical analysis of study data, SPSS 20.0 (SPSS, Inc, Chicago, USA) software was used. The pattern of distribution for continuous variables was determined by a one-sample Kolmogorov-Smirnov test, and data were presented as mean ± standard deviation. If continuous variables were found to be normally distributed, groups were compared by Student’s t-test. When the distribution was not normal, a Mann-Whitney U test was used. Categorical data were given as percentages of the total, and chi-square and Fisher’s exact tests were used. Logistic regression analysis was performed to determine predictors exhibiting a statistically significant relation with AHRE, and these variables were used in multivariate stepwise regression analysis. A p value of less than 0.05 was accepted as significant for all comparisons. To determine the best cut-off values of PQ interval, QTc and diastolic ECG index for predictions of AHRE, a receiver-operator characteristic (ROC) curve was constructed, and the area under the ROC curve was calculated. The value with the highest total sensitivity and specificity was chosen as the cut-off values for prediction of AHRE.

## RESULTS

The study population consisted of 203 patients (mean age: 67.5±9.1, 60.1% male) with dual pacemakers who were divided into two groups with regards to the presence of AHRE in their pacemaker interrogations. Fifty-one (25.1%) patients with AHRE were defined as group 1 and 152 (74.9%) patients without AHRE were defined as group 2. Demographic, clinical, laboratory and echocardiographic data regarding both groups are given in Table 1. The demographic characteristics and cardiovascular risk factors were comparable between the two groups. Echocardiographic variables, including left ventricular end-diastolic and end-systolic dimensions, ejection fraction, wall thickness and left atrial diameter were also comparable between the two groups. Serum sodium levels were significantly higher in patients with AHRE. Serum potassium and serum calcium levels were similar in both groups (Table 1).

Mean heart rate was higher in group 1 as expected, but the difference did not reach statistical significance. Tend-Q was significantly longer in group 2 [401.6±18.1 millisecond (msec) vs. 504.0±27.1 msec, p<0.001]. Also, Tend-P was significantly longer in group 2 (253.4±16.9 sec vs. 370.9±18.8 msec). According to PQ interval (166.3±9.5 msec vs. 147.5±11.0 msec), there was statistically significant prolongation in group 1. The corrected QT interval was significantly longer in group 1. Similarly, QRS duration was found to be significantly longer in group 1 patients (Table 2).

**FIG. 2.**
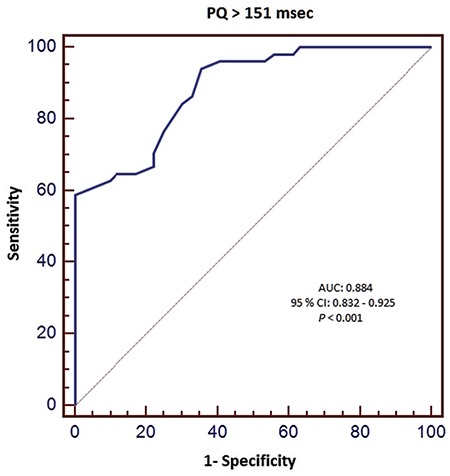
Receiver-operating characteristic curve of PQ interval as predictor of AHRE.

**FIG. 3.**
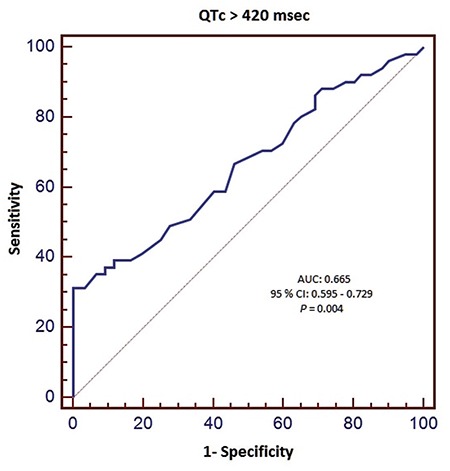
Receiver-operating characteristic curve of QT interval as predictor of AHRE.

**FIG. 4.**
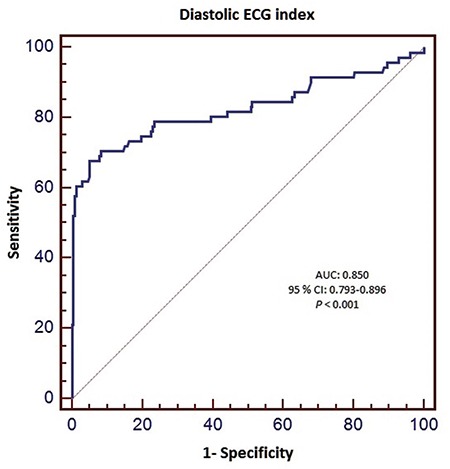
Receiver-operating characteristic curve of diastolic ECG index as predictor of AHRE.

Univariate analysis revealed that diastolic ECG index, serum potassium levels, heart rate and PQ and QT intervals were associated with the presence of AHRE. Diastolic ECG index, heart rate and PQ and QT intervals were the only independent predictors of AHRE in patients with dual pacemakers in multivariate analysis (Table 3). ROC analysis was performed for predictors of AHRE. ROC analysis revealed 58.8% sensitivity and 100% specificity, 100% positive and 87.9% negative predictive values for PQ >151 msec (AUC: 0.884, 95% CI: 0.832-0.925, p<0.001, Figure 2). It also revealed a 38.3% sensitivity and 100% specificity, 100% positive and 81.3% negative predictive values for QTc >420 msec (AUC: 0.665, 95% CI: 0.595-0.729, p=0.004, Figure 3); 76.4% sensitivity and 78.5% specificity, 54.9% positive and 90.9% negative predictive values for diastolic ECG index <0.0342 (AUC: 0.850, CI: 0.793-0.896, p<0.001, Figure 4).

## DISCUSSION

The present study is the first to investigate the association between diastolic ECG parameters and diastolic ECG index with asymptomatic device-detected AF. Our main findings were; 1) ECG diastolic parameters and diastolic index were significantly longer in the AHRE group, 2) Diastolic ECG index, serum potassium levels, heart rate and PQ and QT intervals were associated with the presence of AHRE according to univariate analysis, 3) Multivariate analysis showed that diastolic ECG index, heart rate and PQ and QT intervals were the only independent predictors of AHRE. There have been many technological advances in cardiac pacing, which mostly focused on therapeutic modalities. These include dual chamber pacing and rate modulation. In recent years, cardiac pacemakers have started to be considered as implanted arrhythmia monitors.

In the present study, pacemaker-detected AF was present in 25.1% of patients without previously documented clinical AF and in those who had dual-chamber pacemakers due to SND in 1-year follow-up. In the first 3 months after implantation of pacemakers, Healey et al. (17) detected subclinical atrial tachy-arrhythmias in one tenth of patients and 34.7% of patients had at least one episode of atrial tachy-arrhythmia during a 2.5-year follow-up. Gillis and Morck (18) evaluated 231 patients with pacemakers implanted for SND Atrial arrhythmias were detected in 68% of these. According to The Automatic Interpretation for Diagnostic Assistance study (19) 179 out of 354 (50.6%) patients had AHRE. These results are comparable with our 1-year follow-up results. Cardiac pacemakers have now enhanced monitoring capabilities, and atrial arrhythmias are recognized frequently in patients with pacemakers. These statements raise the question of whether these arrhythmias have any clinical significance. The MOST trial (20) showed that AHRE (atrial rate >220 beats/min for 10 consecutive beats) independently predicted mortality (HR: 2.48) in addition to death or nonfatal stroke (HR: 2.79), and AF (HR: 5.93). The mortality and stroke rate are almost doubled in patients with pacemakers implanted due to SND and who were found to have AHRE during device interrogation than those who were not. The ratio was 6 in the case of developing AF. These findings reveal the importance of AHRE prediction in patients with SND.

DD has significant deleterious effects on atrial mechanics, many of which are pro-arrhythmic. Studies in patients with myocardial infarction (21) or diabetes mellitus (22) have detected an increase in the risk of incident AF in patients with DD. DD is associated with increased stretch in pulmonary veins due to increased left atrial pressure (23). A subgroup analysis of the Framingham Heart Study (12) found a trend towards increased incident AF in patients with trans-mitral peak E/A greater than the median. Namdar et al. (15) have shown that diastolic ECG parameters and diastolic ECG index have valuable accuracy in diagnosing DD.

Tend-P and Tend-Q intervals reflect the electrical timing as well as mechanical diastole. These intervals were found to be shorter in the DD group. Thus, AHRE patients had statistically significantly shorter Tend-P and Tend-Q intervals. The PQ interval has been shown to be an accurate determinant of the definition of atrial contraction timing and in this way, the timing of atrial contribution to late diastolic left ventricular filling as well (24). Consequently, prolongation of the PQ interval leads to the occurrence of an atrial contraction earlier in diastole, which reduces the time of mid-diastolic slow ventricular filling and results in a shorter total diastolic phase in patients with normally functioning ventricles (25). The PQ interval was found to be significantly longer in patients with DD. Also, in our study, the PQ interval was significantly longer in patients with AHRE. ROC analysis showed that PQ >151 msec predicted AHRE with a specificity of 100% and a sensitivity of 58.8%. The AUC was larger for PQ than for the diastolic ECG index. Prolongation of the PR interval was found to be associated with increased AF incidence in the Framingham Heart Study. PQ prolongation shows increased atrial conduction time, which may be related to an increase in AF risk. Atrial electrical and structural remodeling may also be marked by PQ prolongation. The high specificity of the PQ interval in predicting AHRE suggests that patients with a prolonged PQ interval should be further evaluated for AHRE and paroxysmal AF episodes, since AHRE are closely associated with an increased risk of stroke, as some atrial high rate episodes were caused by paroxysmal AF. Nonetheless, AHRE were missed in approximately 40% of the study population when a prolonged PQ interval was used as the sole criterion (low sensitivity), which severely limits the value of this parameter as a screening test. Therefore, a low PQ interval should not be used to rule out AHRE or paroxysmal AF. Wilcox et al. (26) showed an association between QTc duration and echocardiographic DD parameters. They showed that QTc intervals were significantly longer in patients with DD. In our study, the AHRE group also had longer QTc intervals. Namdar et al. (15) showed that diastolic ECG index [Tend-P/(PQ x age)] has the highest specificity and sensitivity in the recognition of DD. The index was significantly lower in DD patients in their study. In our AHRE group, the index was also significantly lower.

The present study was cross-sectionally designed, but it does have its own control arm (patients with no AHRE). The current study was conducted at a single academic center, and thus the results may not be directly applicable to other practice settings. Pacemaker default settings for AF detection were slightly different between manufacturers, which may have resulted in mild ascertainment bias between patients. We used left atrial diameter for the assessment of left atrial enlargement instead of left atrial volume. Finally, in this study, the relation between AHRE and echocardiographic diastolic function parameters, such as mitral valve inflow pattern and lateral and septal mitral annular tissue Doppler velocities were not evaluated. Since all diastolic time intervals on baseline ECGs were measured when the heart rate was between 60 beats/min and 90 beats/min, and the patient was not paced during acquisition of the initial ECG, the cut-off points provided in this study should only be used in patients without bradycardia, tachycardia or pacing. For all diagnostic tests, the diagnostic accuracy of the test depends on the prevalence of the disease in the population studied, according to the Bayes’ theorem. The population in the present study had received a CIED due to an underlying cardiac condition, which increases the prevalence of AHRE compared with the general population. Therefore, the diagnostic accuracy of these parameters to detect AHRE in the general population remains unknown, and the cut-off values found in the present study for various ECG measurements should be interpreted in this context.

In conclusion, abnormal diastolic ECG parameters are powerful predisposing factors for the initiation of incident AF. Both the PQ interval and the diastolic ECG index have an acceptable diagnostic accuracy, as well as a high specificity to predict AHRE. Since AHREs are associated with paroxysmal AF and stroke, patients with a CIED and a prolonged PQ interval or shortened diastolic index should be further evaluated for the presence of paroxysmal AF, and prophylactic anticoagulation should be considered to prevent stroke. Both parameters, but especially PQ interval, lack adequate sensitivity to detect AHRE, so these parameters should not be used to rule out AHRE or paroxysmal AF. Further investigations are required to evaluate the applicability of diastolic ECG parameters in the general population without a CIED.

## Figures and Tables

**Table 1:**
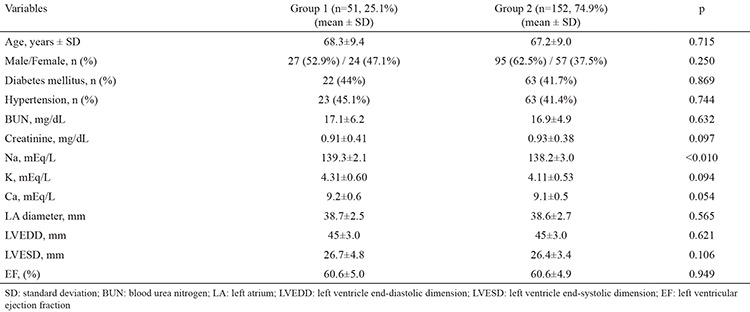
Baseline demographics, serum electrolyte levels and two-dimensional echocardiographic parameters of the 203 study subjects (group 1 and group 2), categorized with respect to the center of origin

**Table 2:**
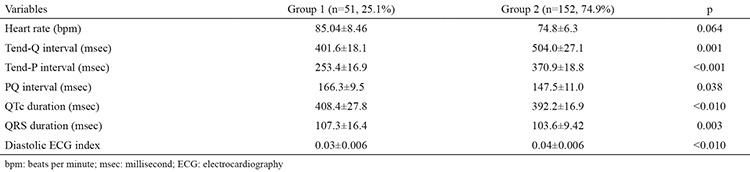
Comparison of electrocardiographic measurements of study population (group 1 and group 2)

**Table 3:**
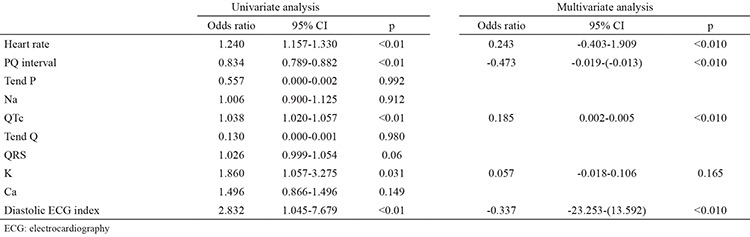
Factors predicting atrial high rate episodes in patients with dual pacemaker on logistic regression analysis
